# Pre-Exposure to an Electrical Stimulus Primes Associative Pairing of Audio and Electrical Stimuli for Dairy Heifers in a Virtual Fencing Feed Attractant Trial

**DOI:** 10.3390/ani10020217

**Published:** 2020-01-28

**Authors:** Megan Verdon, Caroline Lee, Danila Marini, Richard Rawnsley

**Affiliations:** 1 Tasmanian Institute of Agriculture, Faculty of Science, Engineering and Technology, University of Tasmania, Hobart, TAS 7320, Australia; richard.rawnsley@utas.edu.au; 2 CSIRO, Agriculture and Food, Locked Bag 1, Armidale, NSW 2350, Australia; Caroline.Lee@csiro.au (C.L.); Danila.marini@csiro.au (D.M.); 3 School of Environmental and Rural Science, University of New England, Armidale, NSW 2350, Australia

**Keywords:** associative learning, conditioning, cue, priming, shock

## Abstract

**Simple Summary:**

Virtual fencing may soon provide an alternative to electric fencing in livestock production systems. In virtual fencing systems, a collar is worn by each animal and emits an audio cue when the animal approaches a virtual boundary that has been set via a Global Positioning System (GPS). An electrical stimulus is delivered by the collar if the animal continues to walk forward, but not if they stop or turn. Over time, the animal increasingly responds to the audio cue alone. A better understanding of factors that influence learning of the association between audio and electrical stimuli may ensure all animals adapt in systems that utilise virtual fencing. Dairy heifers were reared with or without exposure to electric fencing. Heifers with experience of electric fencing showed more rapid learning of the association between audio and electrical stimuli. There were differences between heifers in the speed of associative learning, perhaps due to individual differences in the significance of the audio cue, the aversive nature of the electrical stimulus, or the animal’s motivation to feed. Ethically acceptable virtual fencing requires that all animals learn quickly how to interact with the technology. The technology and training protocols may require continual refinement to account for individual differences in learning.

**Abstract:**

This experiment examined whether pre-exposure to an electrical stimulus from electric fencing attenuates associative pairing of audio and electrical stimuli in dairy heifers. Two treatments were applied to 30 weaned heifers naive to electric fencing. Heifers in the ‘electric-fence’ treatment were exposed to an electrified perimeter fence and two periods of strip-grazing using electrified poly-wire. Control heifers remained naïve to electric fencing. The pairing of audio and electrical stimuli was assessed in a feed attractant trial using manually controlled training collars. Heifers received an audio stimulus (2 s; 84 dB) when they breached a virtual fence after which a short electrical stimulus (0.5 s; 120 mW) was administered if they continued to move forward. If the animal stopped moving forward no further stimuli were applied. By the third training session, electric-fence heifers received a lower proportion of electrical stimuli than control heifers (*p* = 0.03). The more exploratory interactions a heifer had with the electric fence, the lower the proportion of electrical stimuli she received during training (*r^s^* = −0.77, *p* = 0.002). We conclude that experience with electrical fencing enhanced the salience of the electrical stimulus delivered by manual collars used for virtual fence training.

## 1. Introduction

Electrified poly-tape (i.e., strip-fence) has traditionally been required to intensively graze cattle in pastoral livestock production systems. Strip-fences present a psychological, rather than a physical, barrier. Through a process of avoidance learning, cattle learn to associate the visual stimulus of the strip-fence with an aversive electric shock, leading to active avoidance of the fence. Virtual fencing is an emerging technology that may provide an alternative to electric fencing in intensive pasture-based livestock production systems. This technology uses an audio cue delivered by a collar to signal when the animal nears a virtual boundary that is set via GPS. An electrical stimulus is delivered by the collar if, following the audio cue, the animal walks beyond the virtual boundary [1]. If the animal stops walking or changes trajectory upon delivery of the audio cue then no electrical stimulus is delivered. This type of training is termed positive punishment [2], where administration of the aversive electrical stimulus following the undesired behavior (progressing forward) results in the behavior becoming less likely in the future. Over time the animal learns to associate the audio cue with the pending electrical stimulus unless it changes its behavior, and increasingly responds to the audio cue alone (e.g., Campbell et al. [3]). 

Measures of stress physiology and behaviour indicate that the application of three electrical stimuli at 2 s intervals is no more stressful than common handling events [4]. However, if associative learning of the audio and electrical stimulus is disrupted the animal would perceive to have little control over the receipt of the electrical stimulus, and this apparent lack of control is likely to have consequences for animal welfare [1]. A better understanding of the factors that influence effective conditioning of the audio and electrical stimuli in cattle may ensure all animals adapt quickly to virtual fencing technology, leading to better welfare outcomes [5].

The application of virtual fencing has been well demonstrated on beef breed cattle in extensive grazing systems [3,6,7,8,9,10,11,12], and may enable the implementation of intense and complex grazing regimes in pastoral dairy systems [13,14]. There is considerable variation between individual beef heifers [6,10] and dairy cows [14] in associative learning of audio and electrical stimuli. This variation may be associated with the specific environment and events than an individual has experienced, particularly during the developmental period [15]. For example, dairy cattle are much more likely to have experience receiving an electrical stimulus than beef cattle, through their interactions with electric fencing. Decades of research in laboratory rodents demonstrate that pre-exposure to an electrical stimulus before it is used in associative learning retards conditioning [16,17,18,19,20]. We thus hypothesise that previous experience with an electric fence attenuates associative learning of the pairing of the audio and electrical stimuli in dairy cattle and can account for individual differences in learning. 

In addition to prior experiences, temperament may affect cognitive processes through its influence on emotional state, and consequently attention, memory and judgement [15,21]. For example, fearfulness (i.e., high emotional arousal to a challenging situation; Finkemeier et al. [22]) is a feature of animal temperament [23,24,25] that has been associated with impaired learning in calves [26]. Fear can be induced by unpleasant events that are sudden, unfamiliar, unpredictable and inconsistent with expectations [27]. As such, startle tests have been used to measure underlying temperament as a fear response in dogs [28], calves [29,30] and horses [31]. Interactions with a novel object is another behavioural measure of fearfulness in dairy calves; heifers with a longer latency to contact a novel object have higher cortisol concentrations following the test [25], and administration of an anxiolytic drug increases the time heifers spend in contact with a novel object as well as the post-test decrease in plasma cortisol concentrations [32]. Our secondary hypothesis was that individual variation in fearfulness, assessed in a startle and novel object test, would be related to individual variation in the speed of associative learning.

Using dairy cross heifers in a feed attractant trial, this experiment aimed to determine the effects of pre-exposure to an electrical stimulus from electric fencing on (1) the efficiency of associative pairing of a benign audio cue with an electrical stimulus, and (2) the behavioural response to the audio and electrical stimuli. Relationships between behaviour in a startle and novel object test and that during associative learning were also examined. We predicted that experience with electric fencing would impair associative learning of the audio and electrical stimuli.

## 2. Materials and Methods 

### 2.1. Ethical Statement

All animal procedures were conducted with institutional ethical approval obtained prior to the start of the experiment (University of Tasmania Animal Ethics Committee, A0016940).

### 2.2. Animals and Housing

This experiment utilised 30 weaned dairy cross heifers (Friesian cross Hereford *n* = 15; Jersey cross Hereford *n* = 7; Friesian cross Jersey *n* = 6; Friesian cross Jersey cross Hereford *n* = 2) aged approximately 100 days at the start of the 114 d experiment. Heifers were separated from their dams at birth and housed in semi-enclosed pens (three walls and a roof; 3.5 × 7 m) of 10 to 12 animals until weaning (approximately 60 days of age). At 1400 h on the day of weaning, heifer calves were re-located by truck to one of two adjacent paddocks (140 × 70 m; *n* = 15 heifers per paddock) which were located at the same farm as the rearing pens (approximately 800 m distance apart). The perimeter fence of both paddocks was able to contain heifers without electrification but were also able to be electrified. The assignment of heifers to paddocks ensured the two paddocks were balanced for age (mean ± standard deviation (SD) of 59.5 ± 7.4 days old), breed, weaning weight (mean ± SD of 95 ± 17.6 kg) and the coefficient of variation in weaning weight (18%). Pasture was supplemented with silage when required. Water was supplied ad libitum.

### 2.3. Experimental Design

**Figure 1:**
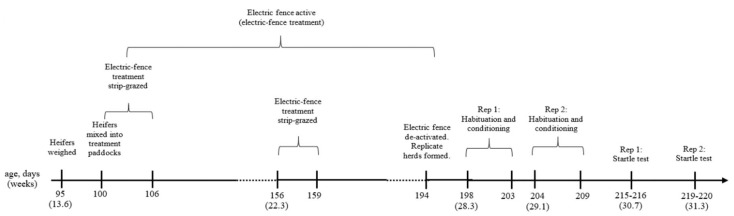
Experimental timeline showing the mean age of heifers in days (with mean age in weeks presented in parenthesis) at which events occurred.

The experimental timeline is summarised in Figure 1. One of two treatments were applied to each of the two groups 15 heifers. The electric fencing for the paddock housing heifers in the control treatment remained off for the duration of the experiment (no experience with electric fencing; labelled ‘control’). Three sides of the perimeter of the paddock housing heifers in the other treatment was electrified for the duration of the experiment (exposure to electric fencing, labelled ‘electric-fence’). The fourth perimeter side separated the two treatment paddocks and was not electrified. To increase the likelihood of interactions with the electric-fence, heifers in the electric-fence treatment were also strip-grazed with electrified poly-tape for the first 6 days immediately following their introduction to their treatment paddock and for 2.5 d 8 w following introduction to their treatment paddock.

To confirm that experimental conditions were imposed on each animal in the electric-fence treatment, eight cameras (MOVII Neo Stream; TechBrands, Guangdon, China) were positioned in-field during the periods of strip-grazing and recorded heifer interactions with the electrified strip-fence during daylight hours (approximately 0500–2130 h, depending on lighting conditions). The cameras were positioned 5 m directly in front of the electric fence which allowed for 10–12 m of the electric strip-fence to be captured in the cameras field of view, with some overlap between cameras. Identifying symbols were sprayed on the flanks and back of heifers with stockspray prior to strip-grazing to allow for individual identification. A total of 67.5 h of recordings were made over the first six days of strip-grazing (average of 11.25 h per day). The electrified poly-tape and cameras were shifted ~5 m every second day and individual markings re-sprayed. During this period, paddocks contained an ample quantity of pasture during (>3000 kg DM/ha), so heifers were not feed-restricted and no supplementation was required. After six days of strip-grazing, the strip-fence and cameras were removed giving heifers access to the full paddock, however, the perimeter of the paddock remained electrified. These procedures were repeated for the 2.5 d strip-grazing period 8 weeks later (a total of 25 h video recordings, an average of 10 h per day). 

Perimeter electric fencing was de-activated for the electric-fence treatment 95 days after heifers were introduced to the treatment paddocks (approximately 14 w). Heifers from both treatments were then recombined to form two new herds of animals comprising electric-fence and control heifers in a 50:50 ratio. From here on, these new herds are referred to as time replicate 1 (*n* = 16 heifers; 14 heifers that underwent training and two spare heifers) and time replicate 2 (*n* = 14 heifers; 12 heifers that underwent training and 2 spare heifers). Time replicates were balanced for age (201 ± 8 d), breed, weight at mixing (167.6 ± 28.6 kg) and the coefficient of variation in weight at mixing (17.1%). 

For logistical reasons, training commenced 4 days after the formation of the new herds for replicate 1, and nine days after the formation of the new herds for replicate 2. Heifers remained in their replicate herds until being subjected to the startle test 11 days following the completion of training (see Section 2.5).

### 2.4. Training of Pairing of Audio and Electrical Stimuli

#### 2.4.1. The Collars 

Electronic collars were used to remotely deliver the audio cues and electrical stimuli. The collars were based on those used for dog training (ET300 Mini-educator, E-Collar Technologies, Garrett, IN, USA), fitted into a custom casing (MooMonitor^+^, Dairymaster Inc., Kerney, Ireland) and enabled an operator to manually deliver audio or electrical stimuli through a remote control device. The range of the collar and remote control device communication system was 800 m. The electronic collar was secured around the neck of the heifers and electrodes that delivered the electrical stimulus were positioned to contact the skin in a shaved area behind the poll. The audio stimulus was a constant polyphonic tone (84 dB) delivered from two speakers attached to the collar just behind the ears of the animal. The electrical stimulus intensity was set to 3 V (120 mW), which equated to a setting of 50 on the 100-point scale provided with the remote control device. The intensity of this electrical stimulus is less than that utilised by others (e.g., 250 mW Lee et al. [4,6,9]), but successfully changed the movement of 6-month old dairy calves toward fresh pasture in a pilot study (evidenced by animals turning away from their direction of movement, stopping or backing up). 

#### 2.4.2. The Test Arena 

**Figure 2:**
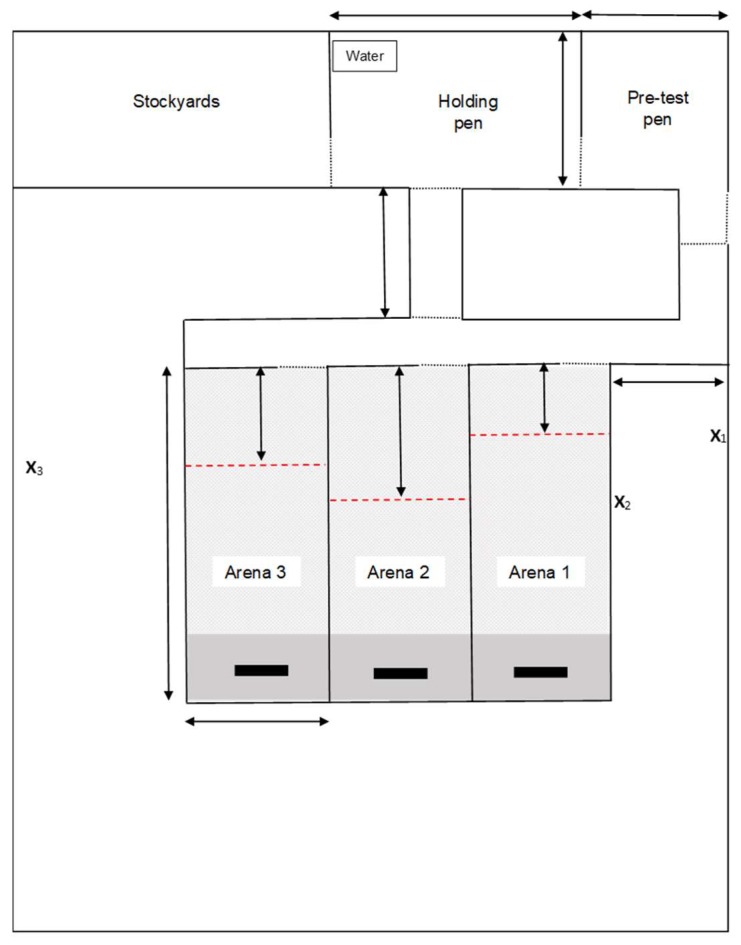
Layout of the associative learning test arena. Use of the three test arenas rotated per training session. Collars were fitted in the stockyards. Individual heifers were removed from the pre-test pen for testing and housed in the holding pen after testing. The exclusion zone was set at a different length for each test arena as indicated by the red dashed line (---). The placement of feed attractant is indicated by black rectangles (**▬**). Gates are represented by a dotted line (⸱⸱⸱). Pasture was mown for the first 90% of each arena (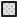) leaving longer pasture as an additional attractant at the far end (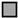). The position of video cameras and of the researcher responsible for administering the audio and electrical stimuli are indicated by X_1_, X_2_ and X_3_ for arenas 1, 2 and 3 respectively.

The test arena where animals were trained to learn the association between the audio and electrical stimuli was purposely built for this research using non-electrified permanent fencing. It consisted of stockyards, two temporary holding pens, and three training arenas (Figure 2). A trough of grain was positioned at the end of the training arena to motivate the animals to move down the far end of the paddock. To further encourage heifers to move to the far end of the paddock, pasture was mown so that only the final 10% of the paddock area contained fresh pasture.

Animals were relocated from their paddock to the stockyards at approximately 0900 h for the fitting of the electronic collars and individualised marking of both flanks using stockmarker. Heifers were then held as a single group in the pre-test pen. After each habituation or training session, heifers were moved to the post-test holding pen where they remained until all animals had been tested (specific habituation and training procedures described in the following sections). Animals were able to graze available pasture in the pre- and post-test holding pens (<1800 kg DM/ha), and water was provided ad libitum. Collars remained fitted for the two habituation or training sessions held each day (session 1 between 1000 and 1100 h, session 2 between 1430 and 1530 h). At approximately 1600 h, heifers were moved back to the stockyards where collars were removed before animals were returned to their paddock (<100 m away).

#### 2.4.3. Habituation Procedures 

Heifers underwent a 3 d habituation period prior to training to familiarise them with the test areas and the location of the feed attractant. The electronic collars were not activated during the six habituation sessions (2 per d). Heifers were introduced to the training arenas in groups of five for the first habituation session, in pairs for the second and individually for habituation sessions 3 to 6. The training arena being utilised was rotated with each session, ensuring that heifers received two habituation sessions per paddock (one AM and one PM). Heifers were given free access to the training arena during habituation and provided with as much time as required to start consuming the grain. Once feeding commenced, heifers were permitted to feed for 3 min. By the final habituation session, all animals began consuming grain in a median of 37 s (range 15–77 s) following entry to the training paddock. 

#### 2.4.4. Training Procedures 

Six sessions of training of the pairing of the audio and electrical stimuli with activated collars were held over 3 days immediately following the habituation period. In replicate 2, low pasture availability meant that heifers were provided with fresh silage between the third and fourth training sessions. 

Individual heifers were introduced to the test arenas for each of the training sessions. The training arena being utilised followed the same rotation as that used during habituation. A virtual fence boundary separating an ‘inclusion zone’ (i.e., area in which animals could move freely) from an ‘exclusion zone’ (i.e., an area beyond which the audio and electrical stimuli would be applied) was established at either 12, 16 or 20 m from the entrance to the training paddock, depending on the paddock being utilised. Distances varied between arenas to delay animals learning an association with the location of the exclusion zone. A researcher with experience in using the manual collars for training heifers was located approximately 20 m outside the training arena to administer audio or electrical stimuli remotely (Figure 2). There was no visual indication of the exclusion zone apart from a small amount of white tape on the fence to aid the researcher.

The following procedures determined the application of the audio and electrical stimuli by the researcher and were adapted from the research using automated virtual fencing collars [3,10]. Based on the researcher’s visual estimation, the audio stimulus was applied for 2 s as the heifer entered the exclusion zone. If the heifer stopped moving further into the exclusion zone, the application of the audio stimulus immediately ceased. If the heifer continued to move forward, however, an electrical stimulus (<0.5 s) was immediately administered by the researcher. If the heifer recommenced or continued movement into the exclusion zone after the delivery of the electrical stimulus, the audio stimulus was re-applied, immediately followed by another electrical stimulus if again she continued to move into the exclusion zone. No further stimuli were applied to an animal in the exclusion zone unless she was further proceeding into the exclusion zone. The training session concluded if (1) no breaches into the exclusion zone were made within 3-min of entry into the training arena, (2) a period exceeding 3 min separated two breaches into the exclusion zone, or (3) a maximum number of 5 electrical stimuli were delivered. Heifers were videotaped during training (Panasonic camcorder, model NV-DS60; Panasonic Corporation, Osaka, Japan) so that their behaviour could be translated at a later date.

### 2.5. The Startle Response Test 

**Figure 3:**
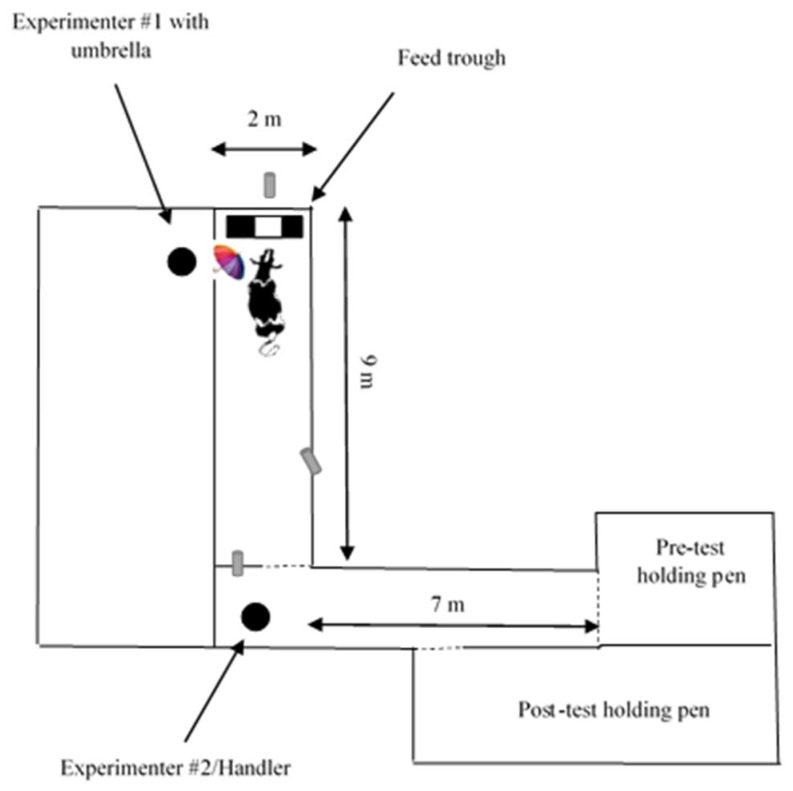
Diagrammatic representation of the startle response test. The walls of the startle response test arena were blackened out to a height of 2.7 m. Three cameras recorded behavioural response from above the test arena at locations indicated by grey cylinders.

The response of heifers to the sudden opening of a brightly coloured umbrella was examined 11 days after the training sessions were completed. The startle response test arena was purpose-built and located inside an empty barn. It consisted of 2.7 m solid panel walls constructed from steel gates and black corrugated polypropylene sheeting (i.e., corflute^®^; Figure 3). A trough of grain (5 kg of Veanavite^®^ weaner calf pellets, 12.0 MJ ME/kg, 18% crude protein) was positioned at the far end of the test arena. The 1.5 × 0.5 m feeding area provided by the trough was covered with corflute^®^ so that animals could only access a feeding area of 0.5 × 0.5 m in the centre of the trough. A small rectangular flap (1 × 5 cm) was cut in the corflute^®^ so that heifers could be observed during the test. The walls of the test arena had 30 cm markings, indicating 0 m at the trough to 9 m at the opposite end of the test arena. Prior to testing, heifers were held as a group in a pre-test holding pen located 7 m from the entrance to the test arena. After testing heifers were moved to a post-test holding pen. No feed was provided in the pre or post-test holding pens, but water was provided ad libitum in both areas. 

Individual heifers were introduced to the startle test arena on four occasions over two days. A morning session commencing at 0930 h and an afternoon session commencing at 1200 h were held on each day. The first three sessions were to habituate heifers to the test arena and train them to the location of the feed. The startle response test was conducted in the fourth session. 

During habituation, heifers were introduced to the startle test arena individually and provided a maximum of 3 min to commence feeding from the trough at the far end of the arena, after which they were permitted to feed freely for 30 s. If heifers did not feed within 3 min, a handler entered the pen and gently encouraged the animal to the trough. The aim of this was to alert the animal to the feed attractant. If the heifer attempted to move around the handler or was hesitant she was permitted to exit the arena. Heifers were moving to the feed trough in a median of 7 s (range 4 to 103 s) by the final habituation session, not including 3 heifers that did not approach the feed in the final habituation session and were therefore excluded from analysis.

The fourth and final session proceeded similarly to the habituation sessions. Heifers were permitted to move to the trough in their own time and to eat freely for 30 s before an experimenter, hidden behind a wall, pushed the umbrella through a small hole in the wall of the startle test and opened it near the heifer’s head (Figure 3). Two heifers consistently fed in bouts of duration <30 s during habituation, so for these animals the umbrella was opened after 15 s of feeding. The umbrella was held in the open position for 2 min before the test concluded. The behavioural response of heifers in the startle response test was video recorded (MOVII Neo Stream; TechBrands, Guangdon, China).

### 2.6. Measures Recorded

#### 2.6.1. Interactions with the Electric Fence 

For heifers in the electric-fence treatment, the number of interactions with the electric fence during the first and second periods of strip-grazing was obtained from video records by a single observer. An interaction was characterised as the receipt of a shock delivered from the fence, visually determined based on physical contact with the fence followed by an adverse behavioural reaction (retreat from fence, shake head, cessation of previous activity along with a rapid postural change, jump or vocalisation (i.e., startle, Taylor [33])). Each interaction was also classified as being exploratory (i.e., a deliberate act from the animal such as sniffing or licking the fence) or accidental (i.e., contact was made while grazing, or if the animal was knocked or fell into the fence). 

#### 2.6.2. Associative Learning

A single observer that was blinded to treatment obtained the following measures from video records taken in each of the 6 training sessions: the time taken to interact with the virtual fence; whether the heifer reached the feed attractant; the time taken for the heifer to reach feed attractant; the behavioural response of heifers to the audio or electrical stimuli (see Table 1 for ethogram). Observations from training session 2 were repeated on a subset of 14 heifers by the original observer and by a second observer. This determined high intra-observer reliability (*r^s^* ≥ 0.93) and inter-observer reliability (*r^s^* ≥ 0.84), although the number of effective behavioural responses to the electrical stimulus had a lower inter-observer reliability than all other classifications (*r^s^* = 0.61). It is recommended that future studies standardise the period following stimulus delivery in which the behavioural response is assessed (e.g., 2 s). The number of interactions with the virtual fence and the number of audio and electrical stimuli delivered were recorded in situ and confirmed using video records. From these data the proportion of electric to total stimuli delivered, and the proportion of effective, ineffective or behaviourally unresponsive reactions to the audio and electrical stimuli, were calculated.

#### 2.6.3. Startle Test 

Withdrawal distance, defined as the furthest distance travelled immediately after the umbrella opened (using the head as a reference point), was determined using the 30 cm markings on the walls of the startle response test arena. This number was estimated via direct observation and confirmed using video records. From video records, data on time taken to reach the feed attractant (interval from the time the heifers two front legs cross the threshold into the startle test to when she lowers her head into the feed trough), return to the feed attractant after the startle (interval from the time the umbrella is opened to the heifer lowering her head into the feed trough), and interact with the umbrella (interval from the time the umbrella is opened to when the heifer physically touches the umbrella with her mouth/tongue/nose/face) were obtained by a single observer. Observations were repeated on a subset of 10 heifers by the original observer and by a second observer which determined high intra-observer reliability (*r^s^* ≥ 0.93) and inter-observer reliability (*r^s^* ≥ 0.97). Heifers that did not return to the feed attractant or interact with the umbrella were given the maximum value of 120 s.

### 2.7. Statistical Analysis

**Figure 4:**
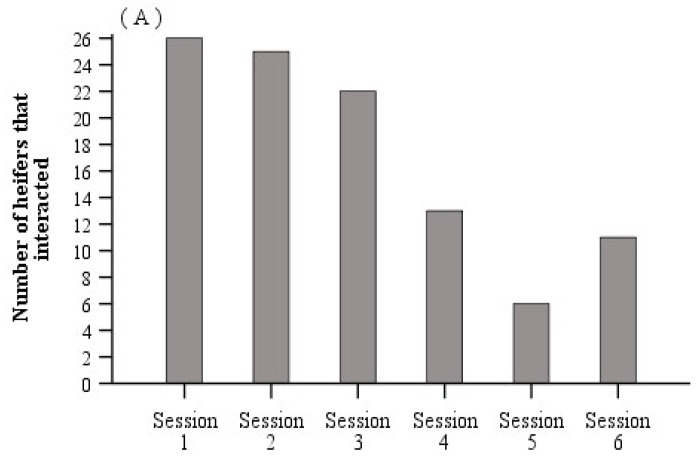

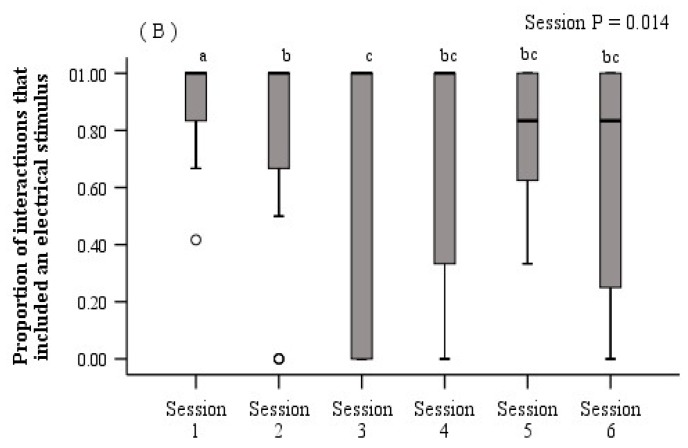
Using data from all heifers and the six training sessions, (**A**) bar chart displaying the number of heifers that interacted with a virtual fence, and (**B**) boxplots of the proportion of interactions with the virtual fence during which an electrical stimulus was delivered. Boxplots show the median and the first and third quartiles (25 and 75% of data), with whiskers extending to the lowest and highest values. Values greater than 1.5 × the interquartile range (IQR) are indicated by օ. Raw means are presented. Estimated marginal means are presented in Table S2. For Figure 4B, training sessions with different superscript letters ^abc^ differ at *p* ≤ 0.05.

All statistical analyses were carried out using the SPSS statistical software package (SPSS 22.0, SPSS Inc., Chicago, IL, USA) and the unit of analyses was the individual calf. From the fourth training session, 81% of heifers could be categorised as consistently avoiding the exclusion zone or consistently tolerating the electrical stimuli to reach the feed attractant (Figure 4, Table S1). As such, the effects of treatment on the proportion of electric to total stimuli delivered was first analysed using data from the six training sessions, and then using data from training sessions 1 to 3. Following this, we determined that data from all six training sessions were not an accurate representation of the efficiency of the associative learning process. As such only data from the training sessions 1 to 3 were used in the analysis of all other variables obtained during associative learning. The significance level α was set at *p* ≤ 0.05 and the α level for tendencies was set at *p* ≤ 0.1.

The effects of treatment, training session and their interaction on variables recorded during associative learning were analysed using generalised linear mixed models (GLMM). Time replicate was included in the model as a random blocking factor. Repeated effects of calf over training sessions were specified using an auto-regression or unstructured matrix covariance, based on the structure associated with the lowest Akaike information criteria scores. Test order was not included in the model as it was not consistent over training sessions (Spearman rank correlations between any two training sessions: *r^s^* ≥ 0.24, *n* = 26, *p* > 0.05). Following visual inspection (quantile-quantile plots and histograms), proportionate data were arcsine square-root transformed and duration data were logarithmically transformed prior to linear analysis, so that residual variation was homogenous between treatments and time replicates. Count data were analysed with a Poisson distribution and log link. Whether or not a heifer reached the feed attractant was binary and analysed using a binomial distribution and logit link. Heifers that did not breach the exclusion zone were recorded as missing values for the following variables: time to interact with the virtual fence or reach feed attractant, behavioural response to stimuli, success in reaching feed attractant (see Table S1 for details on heifers that did not breach the virtual fence). As such, the Satterwaite approximation was used to calculate degrees of freedom. To aid with interpretation, raw means are presented with transformed means (and backtransformed means) ± SEM presented in Table S2.

The startle test was not conducted on four of the 26 heifers (2 from each treatment). One heifer was injured prior to the startle test while the other three heifers would not feed from the trough during the test. Mann-Whitney U-test found no effects of time replicate or treatment on the behaviour of heifers in the startle test (*p* > 0.05), thus animals from both treatments and replicates were pooled for statistical analysis. The relationship between heifer behaviour in the startle test and behaviour during training (totalled over the first three training sessions) were assessed using Spearman rank correlations. For the 13 heifers in the electric-fence treatment, Spearman rank correlations assessed the relationships between the number of interactions with the electric fence (total and exploratory) and (1) the proportion of total interactions with the exclusion zone in which an electrical stimulus was delivered, and (2) the behavioural variables recorded in the startle test.

## 3. Results

### 3.1. Interactions with the Electric Fence

All heifers in the electric-fence treatment interacted with the electrified poly-tape on the first day of strip-grazing. A total of 105 interactions with the electric fence were recorded over the first 6 days of strip-grazing (i.e., immediately following introduction to the paddocks). For individual heifers, the total number of interactions over the six days ranged from 5–10. Forty-seven percent of these interactions occurred on the first day of strip-grazing with 97% of interactions on day 1 being exploratory. By contrast, from days 2 to 6 of strip-grazing there was an average of 11 interactions with the electric fence per day (mean ± SD 0.75 ± 0.73 interactions per heifer and d) and 44% of these were exploratory. There was only 1 interaction with the electric fence during the second period of strip-grazing (imposed 8 weeks after the first period) and this was accidental. 

**Figure 5:**
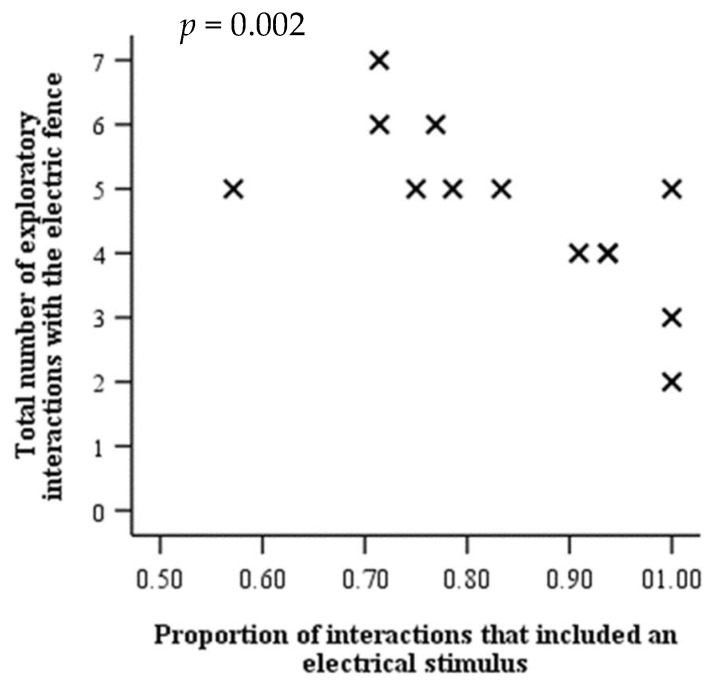
Scatterplot showing the correlation *p*-value and relationship between the number of exploratory interactions with the electric fence per heifer (total over 6 days of strip-grazing) and the proportion of interactions with the virtual fence during associative training that included an electrical stimulus.

The total number of interactions with the electric fence (i.e., accidental + exploratory) was not related to associative learning (Table S3, *p >* 0.05). However, the total number of exploratory interactions with the electric fence had a strong negative relationship with the proportion of interactions with the virtual fence in which an electrical stimulus was delivered (*r^s^* = −0.77, *n* = 13, *p* = 0.002; Figure 5).

### 3.2. Treatment Effects on Associative Learning 

**Figure 6:**
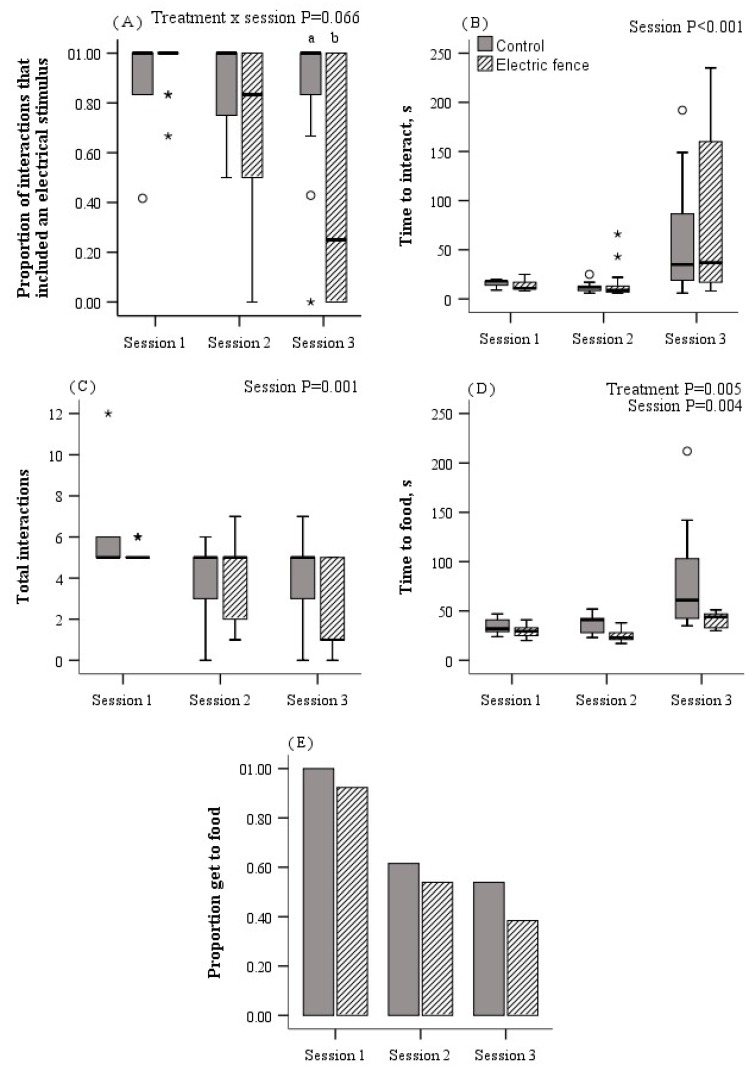
Using data from the first three training sessions, for heifers that had experience of electric fencing (dashed) or no previous experience of electric fencing (control, grey) the (**A**) proportion of interactions with the virtual fence during which an electrical stimulus was delivered, (**B**) time to interact with the virtual fence, (**C**) total number of interactions with the virtual fence, (**D**) time to reach the feed attractant, and (**E**) the proportion of heifers to reach the feed attractant. Raw means are presented. Estimated marginal means are presented in Table S2. Boxplots (Figure 6A–D) show the median and the first and third quartiles (25 and 75% of data), with whiskers extending to the lowest and highest values. Values greater than 1.5 × the interquartile range (IQR) are indicated by օ and greater than 3 × the IQR are indicated by *. In the case of an interactive effect, different superscript letters ^ab^ show where treatment means differ.

The proportion of interactions with the virtual fence in which an electrical stimulus was delivered declined over training sessions 1 to 3 with no further changes between training sessions 4 to 6 (F_5,68_ = 3.1, *p* = 0.014; Figure 4B). There was a tendency for a treatment by test interactive effect (F_5,68_ = 2.0, *p* = 0.092) on the proportion of interactions with an electrical stimulus when data from the six training sessions were analysed (see Table S1 for raw stimulus data from all training sessions). When the analysis was performed using data from the first three training sessions, this tendency for the proportion of interactions with an electrical stimulus to decline over sessions 1 to 3 for electric-fence heifers but not for control heifers further approached significance (treatment × test: F_2,44_ = 2.9, *p* = 0.066; Figure 6A). This interaction was significant (F_2,37_ = 3.9, *p* = 0.03) when animals that never showed a behavioural reaction to audio or electrical stimuli were removed from the analysis on the basis that we could not determine whether associative learning had occurred or not (*n* = 2 animals/treatment). Akaike information criteria scores show that the model fit improved with each manipulation of the data described above. Interestingly, in replicate 2, the proportion of electrical stimuli delivered declined after the provision of fresh silage between training sessions 3 and 4 (see Table S1).

Treatment did not affect the time it took heifers to interact with the virtual fence (F_1,22_ = 0.05, *p* = 0.83; Figure 6B), the total number of interactions (F_1,24_ = 1.08, *p* = 0.31; Figure 6C) or the proportion of heifers reaching the feed attractant (F_1,52_ = 0.25, *p* = 0.62; Figure 6E). However, heifers from the electric-fence treatment reached the feed attractant more quickly than control heifers (F_1,21_ = 9.8, *p* = 0.005; Figure 6D). For both treatments, interactions with the virtual fence declined over training sessions (F_2,49_ = 10.1; *p <* 0.001; Figure 6C), while the time to interact with the virtual fence (F_2,41_ = 27.5, *p <* 0.001; Figure 6B) and the time to reach the feed attractant (F_2,29_ = 14.3, *p <* 0.001; Figure 6D) increased over training sessions. 

### 3.3. Behavioural Response to Stimuli

#### 3.3.1. Behavioural Response to the Audio Stimulus. 

Of the 331 audio stimuli delivered over the three training sessions, 42 effective behavioural responses (13%), 29 ineffective behavioural responses (9%) and 260 behaviourally unresponsive reactions (78%) were recorded. The proportion of effective behavioural responses to the audio stimulus tended to increase over training sessions for electric-fence heifers, but not for control heifers (training session × treatment F_2,22_ = 3.1, *p* = 0.068; Table 2). The proportion of behaviourally unresponsive reactions to the audio stimulus decreased over training sessions (F_2,22_ = 7.9, *p* = 0.003), and did not differ between the treatments (F_1,23_ = 0.16, *p >* 0.05; Table 2). Conversely, the proportion of ineffective behavioural responses to the audio stimulus was lower for electric-fence compared to control heifers (F_1,18_ = 5.1, *p* = 0.036), but did not differ over training session (F_1,15_ = 0.95, *p >* 0.05; Table 2).

#### 3.3.2. Behavioural Responses to the Electrical Stimulus 

A total of 284 electrical stimuli were delivered over the three training sessions. Effective behavioural responses, ineffective behavioural responses and unresponsive reactions were recorded 73, 63 and 148 times, respectively (26%, 22% and 52% of total responses). The proportion of effective responses to the electrical stimulus did not change over the three sessions for control heifers, but electric-fence heifers had a lower proportion of effective behavioural responses in sessions 1 and 2 compared to session 3. Consequently, the treatments differed in the proportion of effective behavioural responses in session 3 (training session × treatment F_1,17_ = 4.8, *p* = 0.021; Table 2). The proportion of behaviourally unresponsive reactions did not differ between treatments in any training session. Experienced heifers were behaviourally unresponsive following the electrical stimulus more often in session 2 compared to sessions 1 and 3, but this proportion was consistent over the 3 training sessions for control heifers (training session × treatment F_2,16_ = 10.8, *p* = 0.001; Table 2). There were no effects of treatment (F_1,58_ = 0.64), training session (F_2,58_ = 0.03) or their interaction (F_2,58_ = 0.60) on the proportion of ineffective behavioural responses to the electrical stimulus (*p* > 0.05; Table 2). 

### 3.4. Relationships to Behaviour in the Startle Test

There were few correlations between heifer behaviour in the startle test and that during training (Table 3). The number of ineffective behavioural responses to the audio stimulus was negatively correlated with the time taken to feed in the startle test, and positively correlated with the time to interact with the umbrella following the startle (Table 3). In contrast, the time to interact with the umbrella following the startle shared a negative relationship of moderate strength with the number of behaviourally unresponsive reactions to the audio and to the electrical stimulus during training (Table 3). For heifers in the electric fence treatment, the total number of interactions with the electric fence (i.e., accidental + exploratory) was not related to behaviour in the startle test, but the total number of exploratory interactions with the electric fence had a moderate positive relationship with withdrawal distance in the startle test (Table 3).

## 4. Discussion

Contrary to expectations, the findings of the present experiment indicate that associative pairing of audio and electrical stimuli is accelerated in heifer calves that have previous experience with electric fencing (i.e., ‘electric fence’ heifers), compared to heifers that had not previously encountered electricity (i.e., ‘control’ heifers). Although there were no effects of treatment on the total number of interactions with the virtual fence during the feed attractant trial, over time electric fence heifers received a lower proportion of electrical stimuli, had fewer ineffective behavioural responses to the audio stimulus, and had more effective behavioural responses to the audio and the electrical stimulus, compared to control heifers. The more intentional interactions a heifer had with the electric fence during the treatment period, the lower the proportion of electrical stimuli she received during training. 

One explanation for the results of the present experiment is that pre-exposure to electrical stimulus via electric fencing enhanced the salience of the electrical stimulus delivered by the collars during training. A highly salient stimulus (i.e., a stimulus that is easily differentiated from all other cues that are simultaneously being perceived) commands attention and facilitates a faster acquisition of training [5,16,18]. Salience can be modified through experience. For example, exposing laboratory rats to circle and triangle shapes without reinforcement in their home cage enhances their ability to discriminate between the two shapes in a task to obtain a feed reward at a later stage [34]. With increased attention to the context in which electrical stimulus was delivered, electric fence heifers may have learned more quickly than they could control the receipt of the electrical stimulus through behavioural change.

The rejected hypothesis of the present experiment, that previous experience with electric fencing would hinder the associative pairing of audio and electrical stimuli, was based on research using laboratory rodents which reported pre-exposure to electrical stimulus retards subsequent learning during Pavlovian conditioning [16,20,35]. As explained by Hall [16], the two most likely mechanisms underlying this ‘pre-exposure effect’ are habituation to the stimulus and the formation of an association between the stimulus and the environmental context which attenuates the establishment of new associations using that same stimulus (i.e., contextual blocking). Differences between (1) conditions in which stimulus pre-exposure occurred and those during training, and (2) the electrical pulse delivered by the electric fence and that delivered by the manual collars (intensity and nature), may have prevented the stimulus pre-exposure effect in the present experiment. 

Treatment was applied to the group in the present experiment. This makes it difficult to distinguish the effects of treatment per se from the effects of the group. Any effect of the group should be minimal, however, because heifers from the two treatment groups were mixed when the time replicate groups were formed and were tested individually.

Variation between individual heifers in associative learning was observed in this experiment. This is consistent with other research that trained 18-month old Hereford [6] and Angus heifers [3] in attractant trials. It is unlikely that this variation was due to poor electrode contact or collar functionality because (1) the fit of every collar was examined before each training session, and (2) collars were swapped between heifers assessed to be of high and low behavioural reactivity, without affecting their behavioural responses. Some variability in both the context that stimuli were delivered and the timing of delivery is expected as the administration of the stimuli is manually controlled. While associative learning occurs best when the conditioned and unconditioned stimulus are presented close together in time, a delay or variability in timing between the two does not prevent conditioning from occurring [36]. Variation in the motivation to access an attractant may affect how willing a heifer is to tolerate the electrical stimulus. For example, heifers are more likely to continue moving forward despite receiving an electrical stimulus when feed is used as the attractant (74% of responses in the present experiment; 56% of responses in Campbell et al. [3]) rather than familiar conspecifics (12% of responses in Lee et al. [6]). In replicate 2 of the present experiment, the proportion of electrical stimuli delivered appeared to decline after the provision of fresh silage. Increased states of hunger may challenge the effectiveness of virtual fencing technology when applied to lactating dairy cattle in intensive pastoral production systems where pasture allocation is often restricted [37]. 

This experiment tested animals individually which does not replicate the conditions that would be experienced by grazing herds of dairy cattle being managed with virtual fencing technology in practice. While individual testing increases scientific understanding of the factors that may affect the adoption of virtual fencing technology, it does not provide an applied understanding of the overall effectiveness of the technology. Recent research has shown that groups of 12 dry dairy cattle being grazed at low stocking densities using virtual fencing technology remain in the inclusion zone 99% of the time [14]. As discussed by Campbell et al. [3], cattle are gregarious species and their behaviour when tested individually may not relate to their behaviour in a herd. Indeed, Campbell et al. [3] found high variability in the effectiveness of virtual fencing between heifers when tested in an individual feed attractant trial, but when virtual fencing technology was applied in a group of six they remained out of the exclusion zone at least 97% of the time. Similar results have been reported in sheep; individually trained sheep have a 48% probability of receiving an electrical stimulus following an audio cue [12], compared to 19% for sheep trained in groups of 5–6 conspecifics [11]. 

An alternative explanation for the individual variation in associative learning observed in the present experiment and proposed by others [3,6] is related to temperamental pre-disposition. In the present experiment, less fearful heifers (indicated by a short latency to interact with the umbrella in the startle test) were more likely to be behaviourally unresponsive to the audio and electrical stimulus, whereas more fearful heifers (indicated by a higher withdrawal distance following the startle) tended to be more likely to show an effective behavioural response to the electrical stimulus. According to the coping styles hypothesis (see Koolhaas et al. [38]), reactive-type animals are more fearful but adaptive to environmental change [39], while proactive-type animals are more likely to be less fearful and rigid in their behaviour [40]. These relationships between associative learning and temperamental pre-disposition may suggest that a proportion of animals could be slower to learn how to interact with the virtual fencing technology, and this may have implications for animal welfare [1,5]. Further refinement of the virtual fencing technology and training protocols may be necessary to adapt the system to account for individual differences in temperament and learning, and ensure its effectiveness when applied to dairy cattle.

The ethical acceptability of virtual fencing technology relies on the ability of all animals to learn the association between audio and electrical stimuli and through this gain a level of predictability and controllability over their environment [1]. Virtual fencing could enable the implementation of increasingly intense and complex grazing regimes in pastoral dairy systems [13,14], and with increased complexity comes a potential risk of some animals not learning to avoid the electrical stimulus [1]. Long-term studies with larger group sizes are needed to determine if all animals are able to learn the association between the audio and electrical stimulus. Research is also required to assess the application and animal welfare impacts of virtual fencing technology when managing livestock in intensive pastoral production systems. We suggest that due to differences in genetics, physiological state, management and life experiences such research needs to be conducted on dairy cattle and intensive pastoral systems specifically, rather than to rely on data produced on beef breed cattle and more extensive systems. 

## 5. Conclusions

To reduce risk to animal welfare associated with virtual fencing, cattle need to be able to learn how to interact with the technology. Results of the present experiment indicate that pre-exposure to electric fencing results in more rapid associative pairing of audio and electrical stimuli during a feed attractant trial. There were differences between heifers in the speed of associative learning, perhaps due to differences in the salience of the audio cue, the aversive nature of the electrical stimulus, or the animal’s motivation to feed.

## Figures and Tables

**Table 1:** Ethogram of cattle behaviours recorded during associative training. Intra-observer reliability *r^s^* ≥ 0.93, inter-observer reliability *r^s^* = 0.61–0.97.

**Time to interact with the virtual fence**
Interval from the time the heifer enters the training arena with two front legs to delivery of the first audio cue
**Reach feed attractant**
The heifer lowers her head into the feed trough located at the far end of the training arena
**Time to reach the feed attractant**
Interval from the time the heifer enters the training arena with two front legs to reaching the feed attractant
**Behavioural response to stimuli**
Stop—within one body length following stimulus delivery, heifer stops moving and with all four feet on the ground remains stationary for a minimum of 2 s
Walk—moving forward one leg at a time with an even gait. Movement continues for more than one body length
Run—moving forward at a pace that is faster than a walk. The head is typically held up. Movement continues for more than one body length
Turn to the side – full body turn of 45–135° so heifer is parallel (or almost parallel) to the virtual boundary
Turn back—full body turn of 135–215° so heifer is facing towards the inclusion zone
Stop feeding—lifts head from the trough/grazing, may also turn or step away from the trough
Shake head—vigorous movement of head and/or neck from left to right
Buck—both hind legs off the ground and extended backwards
**Effective behavioural response**
Stop
Turn to the side, may continue walking parallel to the virtual boundary
Turn back
Turn back and walk/run towards the inclusion zone
At stimulus delivery, stops feeding and doesn’t recommence. May walk back towards the inclusion zone
**Ineffective behavioural response**
Shakes head and/or bucks and/or changes gait from walk to run while continuing to move forward into the exclusion zone
Stops feeding, but recommences feeding within 2 s
**Behaviourally unresponsive**
No discernible change in behaviour (small movements, such as ear twitches, are permitted)

**Table 2:** The effects of treatment (T: experience of electric fencing, ‘Electric-fence’; no experience of electric fencing ‘Control’), training session (S: 1, 2, or 3) and their interaction (T × S) on the proportion of effective, ineffective and unresponsive behavioural reactions to the audio and electrical stimuli. Raw means and pooled standard error (SE_P_) are presented (estimated marginal means are presented in Table S2). In the case of an interactive effect, different superscript letters show where (within training sessions) treatments differ ^a,b^ or (within treatment) training sessions differ ^c,d,e^.

Behavioural Response ^1^	Control			Electric-Fence			SE_P_	*p-*Value		
	1	2	3	1	2	3		T	S	T × S
**Audio Stimulus**										
Effective	0.07	0.14	0.17	0.05	0.32	0.52	0.04	0.07	0.006	0.07
Ineffective	0.09	0.17	0.21	0.07	0.01	0.02	0.02	0.04	0.41	0.23
Unresponsive	0.84	0.69	0.62	0.87	0.67	0.46	0.04	0.70	0.003	0.59
**Electrical Stimulus**										
Effective	0.30	0.29	0.17 ^a^	0.40 ^c^	0.20 ^d^	0.27 ^b,e^	0.04	0.33	0.23	0.02
Ineffective	0.25	0.32	0.34	0.23	0.18	0.10	0.03	0.43	0.97	0.55
Unresponsive	0.45	0.39	0.49	0.37 ^c^	0.62 ^d^	0.63 ^c^	0.05	0.05	0.78	0.001

^1^ Behaviours defined in Table 1.

**Table 3:** Spearman rank correlation coefficients between heifer behaviour in startle test and that during conditioning.

Behaviour during Strip-Grazing or Associative Learning	Behaviour during Startle Test ^1^			
	Time to Feed	Withdrawal Distance	Time to Return to Feed	Time to Interact with Umbrella
Strip-grazing (*n* = 11)				
Total interactions with electric fence	−0.29	−0.20	0.11	0.55 *
Exploratory interactions with electric fence	0.44	0.64 **	0.31	0.03
Associative learning (*n* = 22) ^2^				
Total stimuli delivered	0.06	−0.24	−0.18	−0.40 *
Proportion of electrical to total stimuli delivered	−0.09	−0.09	−0.27	−0.34
Number of time heifer reached feed attractant	−0.15	−0.17	−0.08	−0.15
Average time to reach feed attractant	0.31	−0.02	−0.008	0.22
Frequency, behavioural responses to audio stimulus ^3^				
Effective	0.13	0.10	0.30	0.28
Ineffective	−0.48 **	0.04	−0.05	0.57 **
Unresponsive	0.13	−0.13	−0.18	−0.52 **
Frequency, behavioural responses to electrical stimulus ^3^				
Effective	−0.02	0.38 *	0.003	0.22
Ineffective	−0.08	0.35	−0.10	0.35
Unresponsive	−0.02	−0.29	−0.11	−0.44 **

* Tendency at *p* < 0.1, ** significant at *p* ≤ 0.05; ^1^ Intra-observer reliability *r^s^* ≥ 0.92 ; ^2^ Over the first three training sessions; ^3^ Behaviour classification described in Table 1.

## Supplementary Materials

The following are available online at https://www.mdpi.com/2076-2615/10/2/217/s1, Table S1: The sequence of individual heifer responses to audio and electrical stimuli, Table S2: Estimated marginal means for variables recorded during associative learning, Table S3: Spearman rank correlations between interaction with the electric fence and associative learning.
