# Supplementary material for: Pre-Exposure to an Electrical Stimulus Primes Associative Pairing of Audio and Electrical Stimuli for Dairy Heifers in a Virtual Fencing Feed Attractant Trial
Source: Animals (Basel). 2020 Jan 28;10(2):217. doi: 10.3390/ani10020217 (PMC7070418; doi:10.3390/ani10020217)
Supplement: Supplementary file 1 [file animals-10-00217-s001.pdf]

animals

Pre-exposure to an Electrical Stimulus Primes Associative Pairing of Audio and Electrical Stimuli for Dairy Heifers in a Virtual Fencing Feed Attractant Trial

Megan Verdon [1,*], Caroline Lee [2], Danila Marini [2,3] and Richard Rawnsley [1]

Supplementary Table S1. For individual heifers in the control and electric-fence treatments, the sequence of responses of to the audio cue only (0) and to the audio cue followed by an electrical stimulus (X) across six conditioning sessions held over 3 days (2 sessions per day). Each response indicates an interaction with the virtual fence line. Fresh silage was provided between training sessions 3 and 4 in replicate 2, which is indicated in the table.

Treatment	Heifer ID	Training Session					
		1	2	3 (Silage Provided)	4	5	6
		**Control**					
Replicate 1	1	00000X00XXXX	XXX0XX	000	XXXXX		0XXXXX
	2	XXXX0X		XXXXX			XXXXX
	3	XXXXX	XXXXX	X			0
	4	XXXXX	XXXXX	XXXXX	XXXXX	XXXXX	XXXXX
	5	XX0XXX	XXX				0
	6	XXXX0X	0X0	XX0			
	7	XXXXX	0X		00		00XX
Replicate 2	8	XXXXX	XXXX	XXX		000XXXXX	
	9	XXXXX	XXXXX	XXXXX	XXXXX	0X0	
	10	XXXXX	0XXXXX	XXXXX			
	11	XXXXX	0X0X	XXX0000			
	12	XXXXX	XXXXX	XXXXX	XXXXX	XX0XX0	0X
	13	XXXXX	XXXXX	XXXXX	XXXXX		
		**Electric-fence**					
Replicate 1	14	XX0XXX	XXXXX	XXXXX	XXXXX	XXXXX	XXXXX
	15	XXXXX	XXXXX	0			
	16	XXXXX	XXXXX	X000	X000X00		
	17	XXXXX	00				0
	18	XX0XXX	X0				
	19	XX0XX0	0		0		
	20	XXXXX	XXXXX	XXXXX	XXXXX	XXXXX	XXXXX
Replicate 2	21	XXXXX	XXXXX	XXXXX	X00		
	22	XXXXX	XXXX0X	XXXXX	000XXXXX		
	23	XXXXX	X0XXX0X	0			
	24	XXXXX	XXXXX	XXXXX	XXXXX		XXXXX

25	XXXXX	0	0
26	XXXXX	XXXX0X	0

Supplementary Table S2. Estimated marginal means (with backtransformed means presented in parenthesis) ± pooled standard error (SE_P) for variables recorded during associative learning, including the behavioural response of heifers to audio and electrical stimuli. Data from the first three (1, 2, 3) of six training session were analysed and are presented. Within a row, treatment means ^{a,b} and training session means^{c,d,e} with different superscript letters differ at $p \leq 0.05$.

	**Control**			**Electric Fence**			**SE_P**
	**1**	**2**	**3**	**1**	**2**	**3**	
**Associative learning**							
Sin⁻¹(√Proportion electrical) [1]							
Sessions 1–3 [2]	1.41 (0.97)	1.32 (0.94)	1.28 (0.92)	1.46 (0.99)	1.04 (0.74)	0.71 (0.42)	0.10
Sessions 1–3 (unresponsive heifers removed) [3]	1.39 (0.97)	1.28 (0.92)	1.22 ^{a} (0.88)	1.46^{c} (0.99)	0.93 ^{d} (0.64)	0.51 ^{b,e} (0.24)	0.17
Log₁₀(Time to interact) [4], *s*	1.18 ^{c} (15.1)	1.07 ^{d} (11.7)	1.58 ^{d} (38.0)	1.10 ^{c} (12.6)	1.07 ^{d} (11.7)	1.72 ^{d} (52.5)	0.04
Total interactions	5.79 ^{c}	4.09 ^{d}	3.63 ^{d}	5.25 ^{c}	4.17 ^{d}	2.62 ^{d}	0.25
Log₁₀(Time to food) [4], *s*	1.53 ^{a,c} (33.9)	1.58 ^{a,c} (38.0)	1.83 ^{a,d} (67.6)	1.46 ^{b,c} (28.8)	1.39 ^{b,c} (24.5)	1.62 ^{b,d} (41.7)	0.02
Proportion get to food	0.001	0.39	0.46	0.08	0.46	0.62	0.26
**Behavioural response to stimuli**							
Sin⁻¹(√Proportion responses to audio) [5]							
Effective	0.14 ^{c} (0.02)	0.24 ^{d} (0.06)	0.29 ^{d} (0.08)	0.10 ^{c} (0.01)	0.52 ^{d} (0.25)	0.92 ^{d} (0.63)	0.13
Ineffective	0.17 ^{a} (0.03)	0.29 ^{a} (0.08)	0.45 ^{a} (0.19)	0.15 ^{b} (0.02)	0.03 ^{b} (0.00)	0.03 ^{b} (0.00)	0.07
Unresponsive	1.30 ^{c} (0.93)	1.05 ^{d} (0.75)	0.86 ^{e} (0.57)	1.33 ^{c} (0.94)	1.04 ^{d} (0.74)	0.63 ^{e} (0.35)	0.16
Sin⁻¹(√Proportion responses to electrical) [5]							
Effective	0.51 (0.24)	0.51 (0.24)	0.34 ^{a} (0.11)	0.61 ^{c,d} (0.33)	0.37 ^{d} (0.13)	0.87 ^{b,e} (0.58)	0.08
Ineffective	0.45 (0.19)	0.51 (0.24)	0.51 (0.24)	0.45 (0.19)	0.34 (0.11)	0.34 (0.11)	0.07
Unresponsive	0.74 (0.45)	0.59 (0.31)	0.70 (0.42)	0.59 ^{c} (0.31)	0.85 ^{d} (0.56)	0.37 ^{c} (0.13)	0.13

[1] The proportion of interactions with the virtual fence in which an electrical stimulus was delivered; [2] No further changes from training sessions 4 to 6. EMM (backtransformed means) for training sessions 4, 5 and 6 for the control treatment Control were 1.33 (0.94), 0.85 (0.56) and 0.83 (0.54); and for Electric-fence treatment were 0.67 (0.39), 0.97 (0.68) and 1.03 (0.73); [3] Heifers that never showed a behavioural reaction to audio or electrical stimuli removed from the analysis; [4] Heifers that did not interact with the virtual fence recorded as a missing value; [5] Heifer behavioural response to audio or electrical stimuli. Effective—e.g., heifer turned or stopped moving.

Ineffective—e.g., heifer shook head, bucked, changed pace but continued forward. Unresponsive—no discernible change in behaviour. Intra-observer reliability for behavioural observations was $r^s \geq 0.93$. Inter-observer reliability ranged between $r^s = 0.61$–0.97.

Supplementary Table S3. Spearman rank correlation coefficients between interactions with the electric fence heifer behaviour during associative learning (n = 13).

Behaviour During Associative Learning [1]	Interactions with the Electric Fence [2]	
	Total Interactions	Exploratory Interactions
Interactions with the virtual fence		
Frequency, interactions	−0.45	−0.68 *
Proportion, interactions with electrical stimulus	−0.54	−0.77 *
Frequency, got to food	−0.30	−0.74 *
Average, time to food	−0.13	−0.08
Frequency, behavioural responses to audio stimulus [3]		
Effective behavioural response	0.49	0.70 *
Ineffective behavioural response	−0.06	−0.07
Behaviourally unresponsive	0.21	−0.38
Frequency, behavioural responses to electrical stimulus [3]		
Effective behavioural response	0.003	0.27
Ineffective behavioural response	−0.12	−0.22
Behaviourally unresponsive	0.27	−0.37

*Significant at $p \leq 0.05$; [1] Over the first three training sessions; [2] Over the first 6 days of strip-grazing with the electric fence; [3] Behaviour classification described in Table 1.
